# A portrait of commissioned literature: connecting the dots between medical education and communication companies, authors, funders and publishers

**DOI:** 10.1186/s41073-026-00194-2

**Published:** 2026-05-15

**Authors:** Maud Bernisson

**Affiliations:** 1 Laboratoire Interdisciplinaire Sciences Innovations Sociétés (LISIS), Centre National de La Recherche Scientifique (CNRS), Institut National de Recherche Pour L’Agriculture (INRAE), Université Gustave Eiffel, Université Paris-Est Marne-La-Vallée, Cité Descartes, 5 Boulevard Descartes, Marne-La-Vallée Cedex 02, 77454 Champs-Sur-Marne, France; 2 Institute for Science in Society (ISiS), Radboud University, Nijmegen, Netherlands

**Keywords:** Medical writing, Authorship, Industry-funded research

## Abstract

**Background:**

Medical education and communication companies (MECCs) are key actors in the production of commissioned publications. Analyzing publications that acknowledge medical writing offers a broad overview of their role, which is scarce in the literature. The reason is that activities related to industry-funded research are highly opaque, which makes access to this information particularly hard, and related analyses scarce. This article offers a novel approach to collecting, and thus, analyzing data. It maps how MECCs, funders, authors, and publishers organize to produce this literature and identifies risks of research integrity breaches.

**Methods:**

The database contains the metadata of 29 911 commissioned papers collected from Web of Science (WoS). All articles involving medical writing were collected if they mentioned “medical writing” in WoS, if they were published by MECCs websites, and if they were listed in publication trackers (documents that list the publications of companies, found on the Industry Documents Library (IDL)). The metadata were collected semi-automatically (manually and with R). Reference lists were built to extract MECCs from acknowledgments and harmonize organizations’ names. The analysis maps the relationships among MECCs, authors, funders, and publishers and shows how they connect.

**Results:**

In contrast with pharmaceutical companies and research institutes, only 33.6% of MECCs are listed in the bylines and even less (18%) are acknowledged in the publications found on their websites and on the Industry Documents Library. Even though they provide writing, MECCs are usually not considered as authors.

Medical writing is a flourishing business and MECCs are key actors. Only a few sponsors (5.5%) outsource medical writing to many MECCs, and a few MECCs (7.7%) have a high number of clients. The medical writing market is thus very competitive and can benefit sponsors. MECCs sometimes publish in journals that are owned by their parent or sister companies. The business model of the publishers largely benefits from medical writing by publishing their product, and sometimes providing it as a service.

**Conclusions:**

The database presented in this article offers new routes to explore the key role of MECCs in industry-funded research and to further assess the commissioned literature and its impacts on biomedical science and research integrity.

## Introduction

When a medical writer is the author but not acknowledged as such in a publication, ghostwriting is often considered the core issue. Medical writers can appear in acknowledgments but rarely achieve the status of byline authors in publications. Acknowledgments fail to adequately report the role of each actor involved in the production of scientific communications, but somewhat succeed in reporting the conflicts of interest of researchers as required by most publication guidelines. In its broadly accepted definition, a conflict of interest solely concerns individuals. It occurs when a person “(a) is in a relationship with another requiring the exercise of judgment on the other's behalf and (b) has a (‘secondary,’ ‘unusual,’ or ‘adverse’) interest tending to interfere with the proper exercise of such judgment” [1]. To date, the most effective tools to tackle conflicts of interest revolve around transparency, which is largely insufficient to address risks of integrity breaches stemming from industry-funded research [2, 3].

The organization of industry-funded research enables “institutional practice[s] that ha[ve] epistemic consequences” [4]. Conflicts of interest do not concern just an individual but most actors involved in the production of industry-funded research. A concrete example is the ghost management of medicine [5], which describes how pharmaceutical companies can supervise a broad set of practices and actors involved in the many tasks required for research. Focusing on medical writing provides an opportunity to shed light on these practices. Pharmaceutical companies commission papers either in-house or outsource them to medical education and communication companies (MECCs). MECCs are paid to design and implement scientific communication strategies, ranging from continuing medical education to publication plans. Publication planning involves framing arguments and communicating them through “scientific platforms within the biomedical literature,” which thus includes scientific articles written by medical writers [5]. Clients oversee MECCs, which manage publication plans and researchers who author, legitimize, and bear accountability for the publication. In exchange, researchers benefit from advantages offered by pharmaceutical companies. As a result, the role of the MECC described in the final publication is confined to medical writing, in accordance with the definition of the pharmaceutical and medical writing industries: a medical writer “supports the work of individual publications” [6]. Based on empirical evidence or personal experience, researchers found that medical writing shares the criteria of ghostwriting [7, 8], which is only the tip of the iceberg [5].

Industry-funded research has a structural impact on science, and consequently, its results [9]. For example, industry-funded research is subject to a publication bias, that is, the tendency to publish positive results more often than negative results; in addition, industry-funded research reaches more positive conclusions about the new drug it has developed than independent research [10], a phenomenon also called the “funding effect” [9]. Based on empirical evidence, often discovered in internal documents of tobacco, chemical, food, fossil fuel, and pharmaceutical companies released following US litigation, additional studies have found evidence of several types of “corporate manipulation of research,” which involved medical writing [11, 12]. Analyses of similar types of evidence have revealed practices like sugar-coating drugs in scientific narratives and the scientification of marketing arguments in scientific communication [13–16]. They also illustrate how publication planning intertwines medical writing with marketing [5, 17]. These practices are not only individual but also stem from the organization of the pharmaceutical and medical writing industries to produce scientific communication.

A variety of companies, including publishers or contract research organizations (CROs), have started offering scientific communication services [5], driven by and contributing to the boom in this sector. When a publisher owns a MECC, risks of research integrity breaches increase. For example, a 2009 court case revealed that the *Australasian Journal of Bone and Joint Medicine*, a peer-reviewed journal owned by Elsevier, contained articles that were essentially disguised advertisements for Vioxx (Merck) [18]. Elsevier’s MECC, Excerpta Medica, was found to be involved, which led the publisher to sell it in 2010 [5]. Excerpta is now part of the Adelphi Group, a major scientific communication group. While this is an extreme example, the ownership of a MECC by a publisher, whose business model partly depends on high publication rates, raises concerns about research integrity breaches.

The medical writing industry is a prominent actor in industry-funded research and is thus expected to produce thousands of articles every year. The opacity surrounding its activities makes it difficult, if not impossible, to provide a hard number that represents the extent of the literature this industry produces. A few studies, however, estimate the share of the ghostwritten literature. For example, Gøtzsche et al. [19] selected 44 industry-initiated trials approved between 1994 and 1995 by Danish ethics committees and compared the authors of the research protocols, the individuals who performed the analysis, and the authors of the publications. They found discrepancies, and thus ghost authorship, in 75% of their dataset; Flanagin et al. [20] surveyed authors about their role in writing publications they authored, and found that 11% of the papers published by three main and three smaller biomedical journals in 1996 were ghostwritten. Based on an internal document (a list of publications by a MECC) made available during a lawsuit involving Pfizer, Sismondo [21] found that between 18 and 40% of articles about Zoloft (sertraline) were managed by MECCs, hired by Pfizer. These results are old and vary due to differences in methods and in the scope of the studies, hence the importance of drawing a more contemporary portrait of this type of literature.

This paper offers a new method for investigating sponsored-research literature. It includes the construction of a database of 29 911 references of commissioned papers [22]. Its analysis describes how medical writing configures the relationships of the actors involved in the scientific publication industry. MECCs constitute the core of the analysis, which highlights their relationships with other actors involved in scientific publishing and identifies the risks of research integrity breaches stemming from these relationships. The first inquiry focuses on authorship. The second traces the collaborations between funders and MECCs. The last inquiry features the relationships between MECCs and publishers.

## Methods

The database of commissioned papers encompasses the DOIs of references from online resources: 229 articles from publication trackers (documents listing commissioned publications produced for a company) discovered in the Industry Documents Library^1^ (IDL database); 4 555 publications made available on the websites of MECCs and CROs (MECCs database); and 26 858 articles from Web of Science that mention “medical writ*” in the acknowledgments (WoS database). DOIs of these publications from Web of Science were then collected, if they existed and were indexed, with semi-automatic methods (see Table 1).

**Table 1:** Number of DOIs found in the Industry Documents Library and on the MECCs websites by company

**(a) DOIs found with publication trackers of companies in the Industry Documents Library**		
***Companies***	***#References found***	***#DOIs found on WoS***
McKinsey (Purdue)	51	37
TEVA	125	15
JUUL	24	5
Mallinckrodt	388	146
Philip Morris	138	26
*Total*	*726*	*229*
**(b) DOIs found on the websites of the MECCs that make their publications publicly available**		
RTIHS	8612	2709
Open health	678	557
IQVIA	3599	519
COS	719	361
Cellworks	156	90
GKM	149	80
Kstrategic	48	47
RBCCons	116	47
KJT	57	48
Alcedis	43	32
MC Analytics	38	17
Costello	180	17
Boston	20	16
Gouya Insights	19	15
Csur	21	12
*Total*	*14 455*	*4567*

I downloaded the BibTeX files of the final list of DOIs on Web of Science, and converted these files into a dataframe with the R library Bibliometrix, which resulted in data loss (12 DOIs from the MECCs database). The final database contains 31 642 items, including duplicates, or 29 911 unique DOIs.^2^ 25 423 of the 29 911 unique publications acknowledge medical writing. The earliest paper was published in 1972 and the latest in 2025 (see Figure 6 in Appendix 1).

BibTeX files do not provide a list of MECCs but of acknowledgments. Bibliometrix does not identify MECCs either. I thus built and used a reference list of MECCs to extract the companies mentioned in the acknowledgments of the papers with the R library stringr. The database needed further cleaning, and I built additional reference lists to harmonize the names of publishers, affiliations, and funders in R (see Appendix 2). In addition to Bibliometrix, I used dplyr and tidyr for the analysis, and ggraph and ggplot2 to visualize the results (Appendix 1 describes the method in greater details).

## Results

### Authorship

**Fig. 1:**
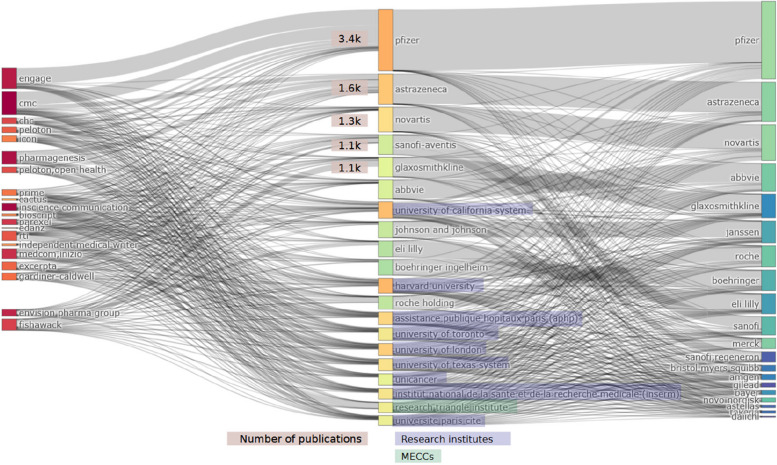
Top collaborations among MECCs (if acknowledged in the publications), researchers (through their affiliations) and funders (threeFieldsPlot() from Bibliometrix 4.3.2)

The first line of inquiry investigates authorship through authors’ affiliations of the papers in connection with their funders and MECCs to explore transparency practices and the distribution of responsibilities among the actors involved in the production of papers. Top funders are pharmaceutical companies. Pfizer sponsored 9.9% of all unique publications (2 975 of 29 911), followed by Novartis, which funded 6.6% publications (*n* = 1 971), and AstraZeneca, which sponsored 6.5% publications (*n* = 1 949). It can either indicate that these companies implement better transparency practices than others, or that they subcontract MECCs more often to produce publications. Pharmaceutical companies often play a larger role than mere sponsors, as suggested by Fig. 1, which visualizes the relations between MECCs, affiliations of (co-)authors and funders in the 25 423 publications acknowledging MECCs and funders.

The figure suggests that pharmaceutical companies’ employees often (co-)author publications sponsored or co-sponsored by their employers, even though MECCs are involved to provide medical writing. The figure also suggests strong collaborations between MECCs and sponsors. MECC employees are clearly under-represented in the affiliations (only Research Triangle Institute stands out), indicating a lack of transparency on the role of MECCs, which mostly appear in the acknowledgments. Excluding the publications found on the WoS from the analysis shows a different dynamic. Only 18% (868 of 4 784) of the publications found in the IDL and on the websites of MECCs mention a MECC in the acknowledgments (see Appendix 1, p9). In addition, MECCs appear in the byline of 33.6% (*n* = 1 609) of these publications, and especially the Research Triangle Institute, which appears in the byline of 1 117 of its 2 709 publications.

The affiliations displayed in the articles of the database highlight and question the role played by research institutes, through their researchers, in contributing to the collaborations between MECCs and sponsors.

**Fig. 2:**
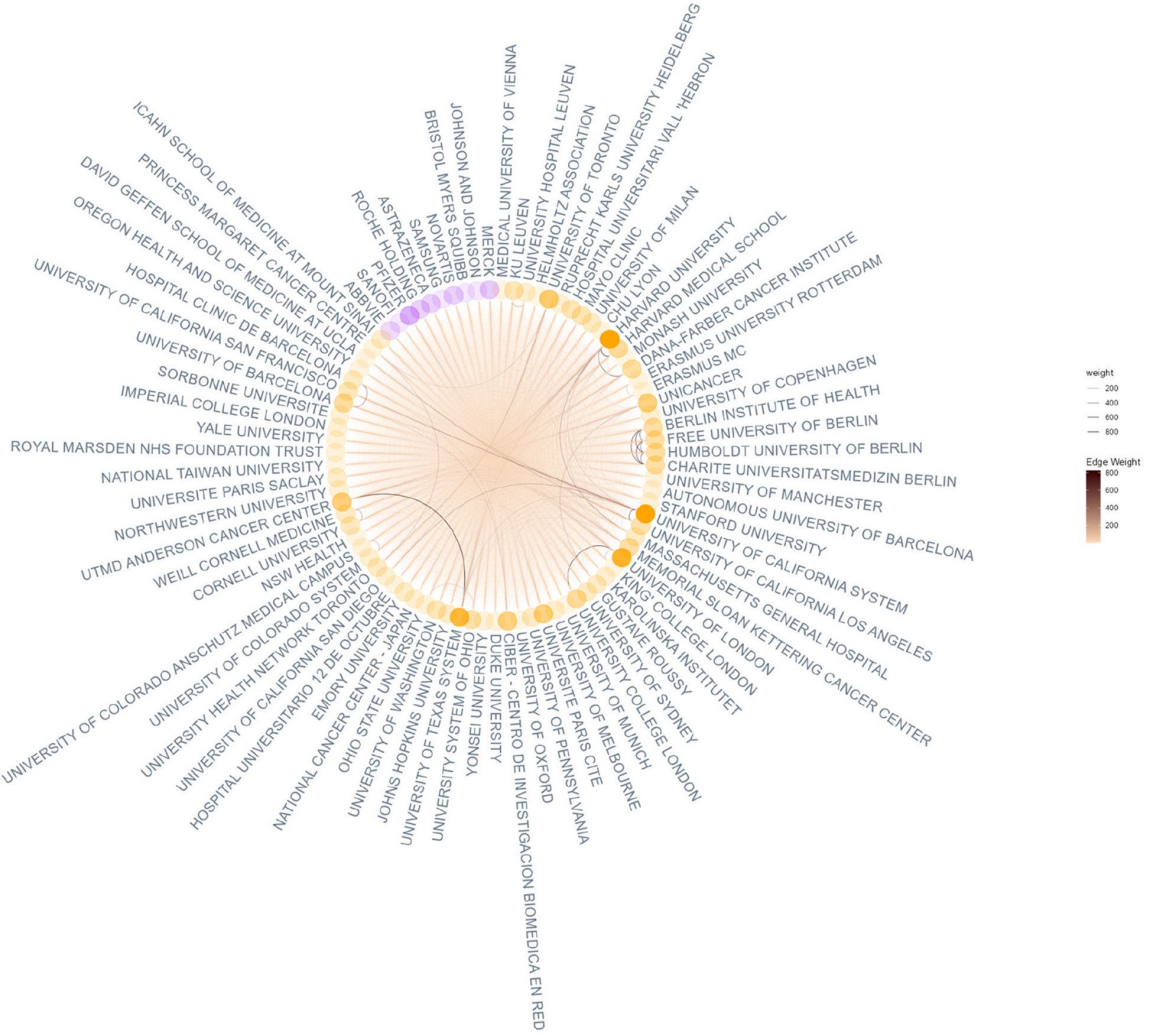
Network of co-affiliations (ggraph 2.2.1)

In Fig. 2, the 83 nodes represent authors’ affiliations that appear more than 4 500 times in co-authored publications (23 753 publications are co-authored). The network includes 10 pharmaceutical companies (“Samsung” refers to “Samsung Bioepis,” specialized in biosimilars) and 73 research institutes, which constitute 88% of the nodes. The most opaque nodes represent the highest numbers of collaborations. In other words, Pfizer’s employees are the most collaborative authors among pharmaceutical companies, while authors affiliated with Harvard University, the University of California System, the University of Texas System, and the University of London, are the highest collaborators among research institutes. The opacity of edges also represents the highest numbers. Edges are distributed evenly between pharmaceutical companies and research institutes, except for a few that tend to collaborate closely, such as Harvard University with Harvard Medical School. However, affiliations in this figure can also belong to the same researcher. The even distribution shows, like Fig. 1, a common use of researchers affiliated with research institutes to author these papers. In addition, no MECC appears in this network of co-authored papers, which, again, questions the potential misalignment between the role performed by a MECC and the role attributed to this MECC in the final paper.

This configuration made of the acknowledgments of MECCs in publications, affiliations of independent researchers and employees of pharmaceutical companies, and pharmaceutical companies as sponsors probably indicates publication planning.

### MECCs and funders

Among the 25 423 publications acknowledging MECCs, one publication can be funded by one or by several sponsors and it can acknowledge the work of one or several MECCs. A methodological approach can thus consider the relationships between individual funders and individual MECCs or the relationships between several funders and several MECCs to produce a publication. The first approach provides insights into the recurrence of collaborations between one funder and one acknowledged MECC across publications. In the database, 1 046 MECCs received funding from an average of 14.4 funders, but with a median of 3. In fact, 81 MECCs (7.7%) received funding from more than 50 different funders (Figure 15 in Appendix 1 visualizes the quantiles of this dataset). Regarding funders, 1 123 of them funded publications acknowledging an average of 13.4 different MECCs, with a median of 3. Only 62 funders (5.5%) funded more than 50 different MECCs acknowledged in publications (Figure 16 in Appendix 1 visualizes the quantiles of this dataset). To summarize, a small group of pharmaceutical companies subcontracted a small group of MECCs.

**Fig. 3:**
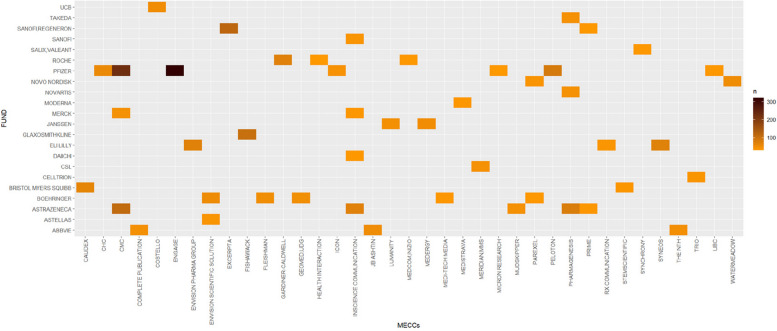
Number of publications by funder(s) and by MECC(s) acknowledged (ggplot2 3.5.1)

This approach fails to represent the rate of production of publications by funders and MECCs because it counts every publication by unique funder and by unique MECC. Thus, if several funders or several MECCs have collaborated, the same publication will be counted several times. For example, Sanofi alone funded 155 publications that acknowledge Excerpta, and Regeneron alone funded 138 publications that acknowledge Excerpta. However, 119 of these publications, which acknowledge Excerpta, are the result of a collaboration between Sanofi and Regeneron. Considering co-funders and MECCs collaborating to produce a publication can overcome this issue. Figure 3 shows the most recurrent co-funding collaborations, such as the collaboration between Sanofi, Regeneron, and Excerpta, and captures rates of publications. Figure 3 represents 2 852 publications (9.5% of the 29 911 unique publications in the database) and displays 47 collaborations between funders and MECCs, which led to the production of more than 30 publications. The MECC InScience Communications, part of Springer Nature Group, has the highest number of clients (4). Pfizer is the company funding the most MECCs in this graph (7). These numbers provide insights on the business models of the MECCs, which seem to depend on a few clients. These findings also suggest a recurrence of collaborations between major pharmaceutical companies and MECCs, which also probably indicates publication planning.

### MECCs and publishers

Mapping the MECCs acknowledged in publications together with the publishers of those publications shows which one publishes commissioned papers the most. MECCs primarily target major and legitimate publishing avenues: Wiley, Elsevier, Springer, and Taylor and Francis are the main publishers of this literature, and some of these publishers belong to the same parent company (see Figure 19 in Appendix 1). For example, Springer Nature acquired BioMed Central (BMC) in 2008. Taylor and Francis acquired Dove Medical Press in 2017 and Future Medicine, part of Future Science Group, in 2023.

**Fig. 4:**
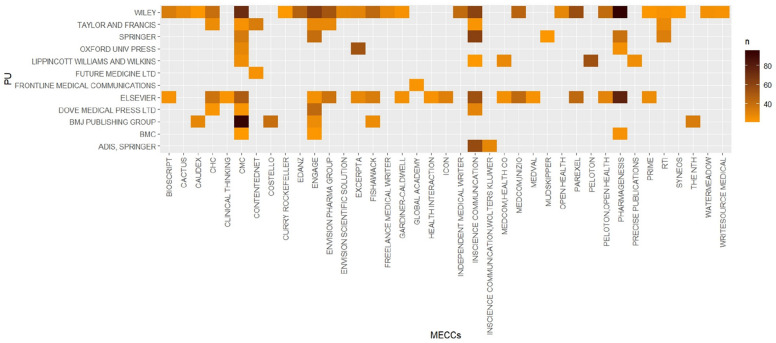
Number of publications by publishers and by MECCs (*n* > 20, ggplot2 3.5.1)

**Fig. 6:**
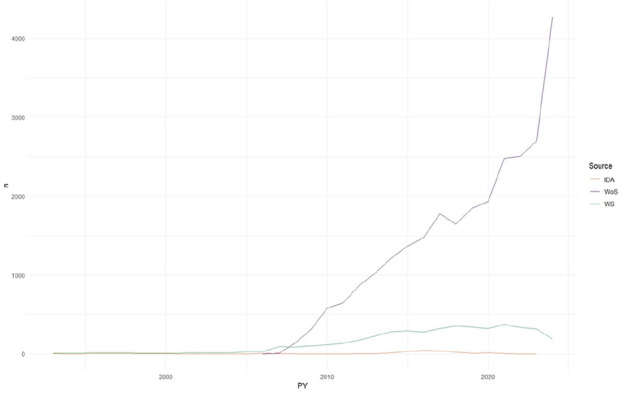
Number of scientific publications in the database by publication year and by source (until 2024)

**Fig. 13:**
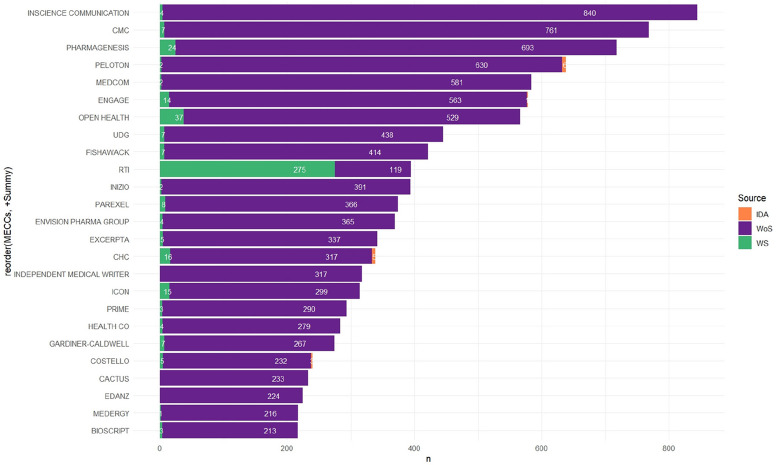
Unique MECC by publication (more than 200 publications)

**Fig. 15:**
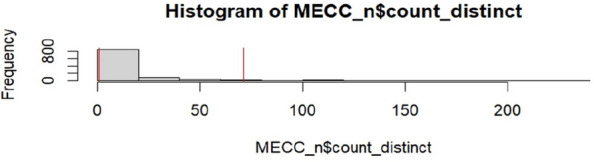
Quantiles of the number of acknowledged MECCs by funder (quantile() function including probs=(c(0.05,0.95)))

**Fig. 16:**
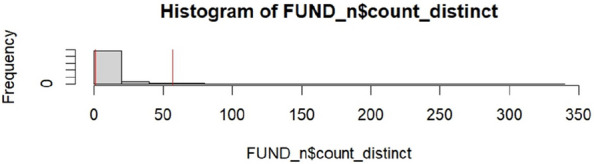
Quantiles of the number of funder by acknowledged MECC(quantile() function including probs=(c(0.05,0.95)))

Figure 4 represents 2 941 of the 25 423 publications acknowledging MECCs. The most acknowledged MECC in this literature is InScience Communications (844), which belongs to Springer Nature (Figure 13 in Appendix 1). Figure 4 shows that it published at least 119 papers in journals owned by its parent company. Collaborations between InScience Communications and Wolters Kluwer led to 29 additional papers published by Adis, Springer. In the same vein, Frontline Medical Communications published 23 publications that acknowledged Global Academy, with which it is currently affiliated. Some MECCs (or larger businesses providing MECC services) also own publishers. For example, Wolters Kluwer currently owns Lippincott Williams & Wilkins.

### Limitations

Analyses involving MECCs only consider publications acknowledging MECCs (25 423), which constitutes a smaller dataset than the total number of unique publications (29 911). The frequent merging, rebranding, and selling of branches of pharmaceutical companies and MECCs limit the possibilities to identify them accurately over time. Even though they play a key role in industry-funded research, key opinion leaders are absent from this study because they are difficult to study across different topics since they specialize in one domain of research. Other limitations include the lack of transparency about medical writing in scientific articles, even though current transparency practices partly permitted this study to be conducted.

## Analysis and discussion

This study provides new insights on the organization of industry-funded research and suggests strong relationships between major MECCs, research institutes, funders, and publishers.

### Transparency and research independence

Collaborations among MECCs, sponsors, and well-known research institutes reconfigure authorship. While MECCs are increasingly acknowledged in publications, they typically do not appear in the bylines, even though previous research indicates that they contribute a substantial portion of the writing, if not all of it [5, 7]. On paper, byline authors are responsible for the content, not medical writers or pharmaceutical companies. The ICMJE’s definition of authorship aligns with industry’s definitions of medical writing, such as the Good Publication Practice (GPP) Guidelines, which state that a medical writer only “contributes” to a manuscript and should be acknowledged as such. This definition frames the problem of medical writing as a lack of transparency, and overshadows key issues related to publication planning such as corporate responsibility [23], conflicts of interest of KOLs, medical writers and company employees [4, 24], and marketing [7, 25, 26].

The overview of the most common affiliations suggests strong collaborations between pharmaceutical companies and research institutes, raising questions about research independence. Representing prominent research institutes, the authors participate in a network made of MECCs and sponsors, which configures the nature of their relationships, whether financial or of another nature. Recurring collaborations among researchers affiliated with research institutes, sponsors, and MECCs have been previously described in isolated cases [7, 8]. The cumulative evidence from these cases [5, 17, 25], combined with these findings, suggests that this is rather common practice. Within this organization, the independence of those researchers is in jeopardy. The organization of actors to publish scientific communication also questions the efficacy of conflicts of interest and transparency requirements, which mainly rely on self-regulation [2, 3]. They fail not only to disclose the extent of the conflicts of interest of those researchers, but also to describe the precise role of the sponsors and the MECCs.

### Financial relationships

The medical writing market benefits pharmaceutical companies, as a few pharmaceutical companies subcontract a select number of MECCs to produce commissioned papers. The market seems to be highly competitive for MECCs. Findings also suggest that the main pharmaceutical companies resort more to MECCs for commissioned papers and might have better transparency practices than smaller companies. However, such conclusions cannot be confirmed unless those companies make their financial relationships and contractual obligations publicly accessible.

Publication trackers and Fig. 3 suggest that the production of articles by batches, or publication planning, is common practice. The publication trackers found in the IDL are a result per se as they trace publications and indicate, in some cases, the product supported by several, if not many, publications (eg, Mallinckrodt’s publication tracker in Appendix 3). Healy & Cattell [27] used a similar document made available during a litigation and authored by a MECC that worked for Pfizer to promote its drug Zoloft (sertraline). They found that the commissioned literature had 50% more impact on the therapeutic domain than the non-commissioned literature on sertraline. The authors underlined that sponsors, or pharmaceutical companies, tend to avoid publishing negative results, and sponsorship has become “a demonstrable predictor of the findings of that study,” a clear example of the “funding effect.” This can also be a sign of disguising marketing as scientific communication. Litigation cases and previous studies found that many companies, including Purdue, [13, 16] Mallinckrodt [28], and Philip Morris [11, 12, 14, 15], have promoted misleading information in scientific publications about the health impacts of their products. Out of 229 of their publications (see Table 1), none were retracted nor received an expression of concern. Further inquiry is necessary to understand these low numbers and the potential impact of these publications on prescribers, researchers, and policymakers.

Relations among sponsors are illustrated through thousands of co-funded publications in the database. The majority of these funders are pharmaceutical companies while government organizations are a minority. Financial involvement of several sponsors to produce research, such as the collaboration between Sanofi and Regeneron to develop a new drug, illustrates how increasingly common collaborations among sponsors have become. However, the difficulty in accessing information about these collaborations limits further analyses [29].

**Fig. 18:**
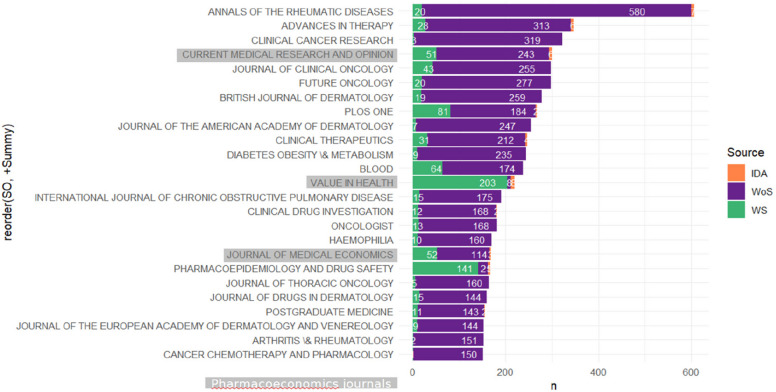
Number of publications by journal (with duplicate DOIs, *n* > 150)

**Fig. 19:**
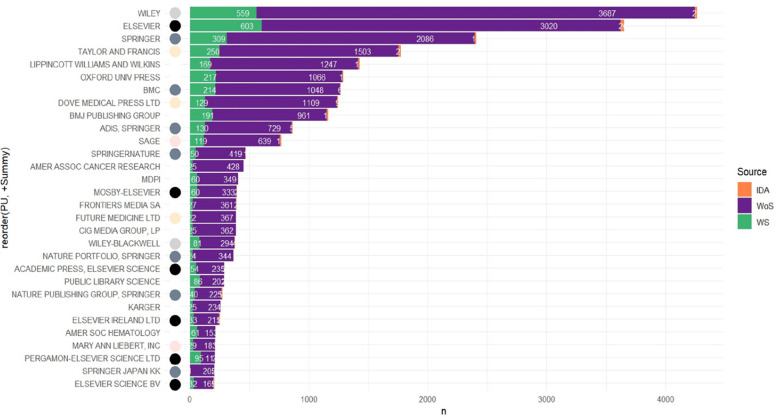
Number of publications by publishers and by source of data collection (*n* > 200, with duplicate DOIs)

The medical writing market highly benefits the business model of for-profit publishers. Authors affiliated with a research institute and who benefit from the financial support of pharmaceutical companies have more chances to publish quickly in costly avenues. Commissioning a manuscript approximately cost $13 375 to $18 000 in the late 1990s [30]. A 2015 proposal submitted by Peloton Advantage to Teva Pharmaceuticals offered “Primary Manuscript development and submission” for $37 924 [31]. In twenty years, prices may have at least doubled. On top of medical writing, authors have to pay Articles Processing Charges (APCs). APCs of high-impact journals often start at $2 000 (median for gold open access in 2023) and can reach $12 290 (*Nature *portfolio of hybrid journals in 2024) [32]. Publishers are developing additional services, which make publishing even more costly and less accessible for independent researchers. For example, big publishers, such as Taylor & Francis (T&F), Future Medicine Ltd. (now T&F), and Elsevier, have developed paid fast-track or “accelerated” publishing services for a selection of journals, so that a publication can be reviewed faster than traditional peer review. In 2022, T&F offered this service for almost double the price of the standard cost of publication [33]. In an article published by the T&F Journal “Current Medical Research and Opinion” (see Figure 18 in Appendix 1), employees of Pfizer, AstraZeneca, Merck, and the MECC MedThink SciCom described how “fast-track review” could make the publication process more efficient [34]. These services and increasing costs of APCs for high impact journals may be aligning with the financial capacities of sponsored researchers, but not with those of independent researchers. Additional financial relationships between pharmaceutical companies and publishers include advertising, database subscriptions, and the sale of reprints.

The medical writing market and the publishing market also overlap. Some companies own both MECCs and publishers, which are indicators of vertical integration and a shift towards an oligopoly. This configuration, as for example the ownership of both a MECC and a publisher, such as InScience Communications and Springer Nature, increases the risks of integrity breaches. Findings show that MECCs sometimes publish in journals owned by a publisher that is either a sister or a parent company. This configuration can jeopardize the independence of journals and editors, as demonstrated by the case involving Elsevier, its previously owned MECC Excerpta, and Merck [18]. Another example involves Informa, which owns Routledge and Taylor & Francis. In a 2015 email, the publisher offered discounts for pre-prints and perpetual open access to the opioid manufacturer Mallinckrodt in exchange for the publication of 15 manuscripts in five of its journals [35]. Among them were listed *Postgraduate Medicine*, *Current Medical Research & Opinion*, and the *Journal of Medical Economics,* which are also among the top publishers of the commissioned literature that is constitutive of the database (Figure 18 in Appendix 1).

## Conclusion

The nature, often financial, of the relationships between publishers, pharmaceutical companies, research institutes, and MECCs alerts about the risks of integrity breaches that can stem from their collaborations. The findings in this article highlight these issues and offer new routes to explore the key roles of MECCs, even though such inquiries are inevitably limited due to the opacity surrounding their activities. They confirm that conflict of interest disclosure is not enough to address the systemic problems of corporate-sponsored research.

This study also supports the claim that publication planning is common [5]. The relationships among actors hired by pharmaceutical companies to produce and circulate scientific information create a nexus of conflicts of interest conceptualized as “institutional corruption” [4] or the “ghost management” of medicine [5]. Ultimately, readers bear the burden of identifying potential conflicts of interest and biases in peer-reviewed articles.

Descriptions of systemic problems also tend to disregard publishers. As discussed, the medical writing industry and the publishers have developed services partly or fully for pharmaceutical companies. Their business model thus depends or thrives on the needs of their clients.

More stringent regulations and transparency measures about the relationships between those actors are crucial. The database made available through this study seeks to be part of this solution by increasing transparency on their relationships, and offering possibilities for further assessing the literature produced by these collaborations and their impacts on biomedical science and research integrity.

## Figures and Tables

**Fig. 5:**
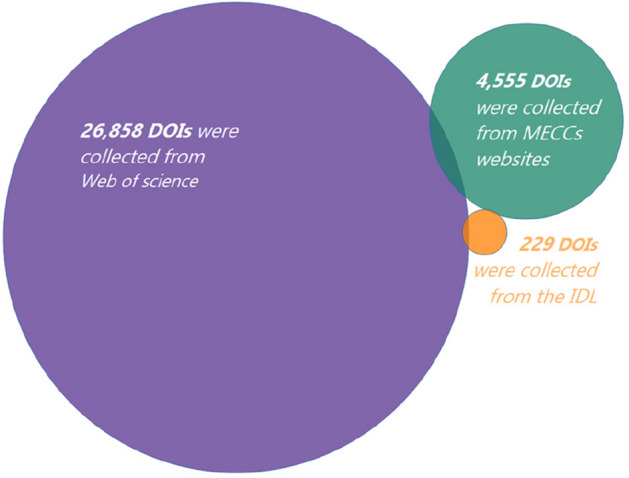
Number of scientific publications by source of collection, overlaps of different sources indicate duplicates

**Fig. 7:**
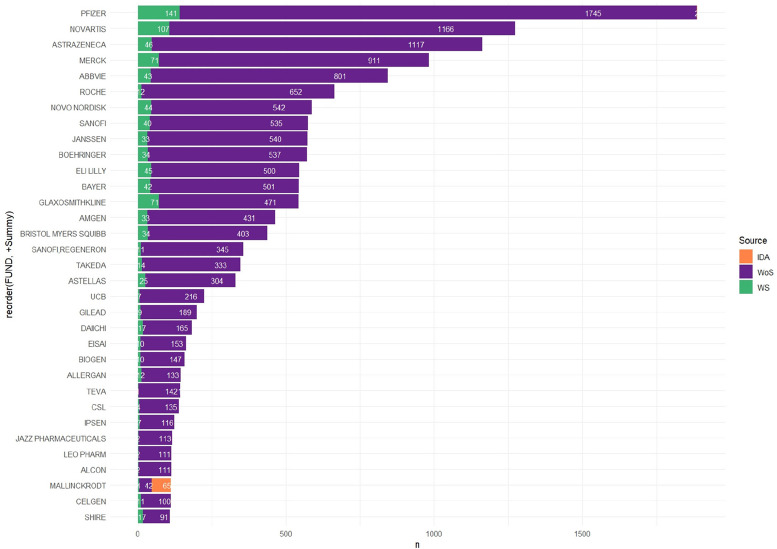
Funder and co-funders by publication (more than 100 publications)

**Fig. 8:**
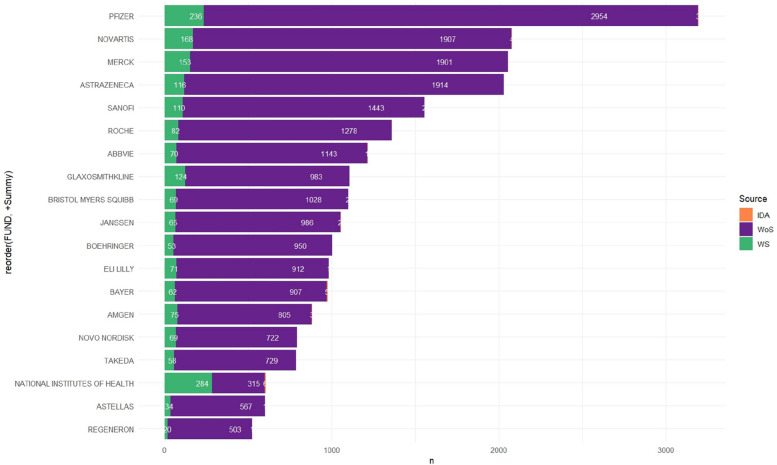
Unique funder by publication (more than 200 publications, exclude co-funders)

**Fig. 9:**
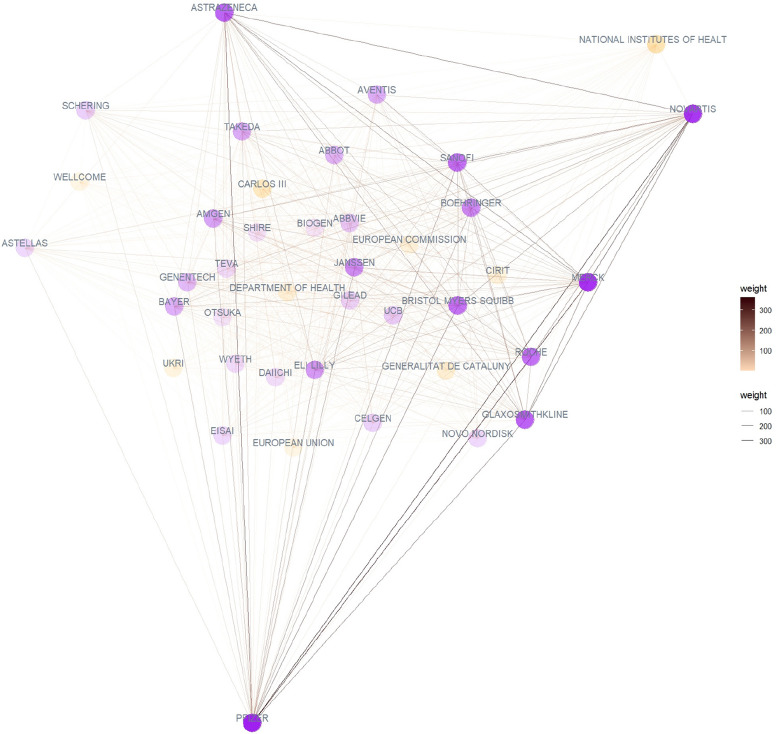
Network of co-funders (vertices degree > 1000, without duplicates, 40 nodes)

**Fig. 10:**
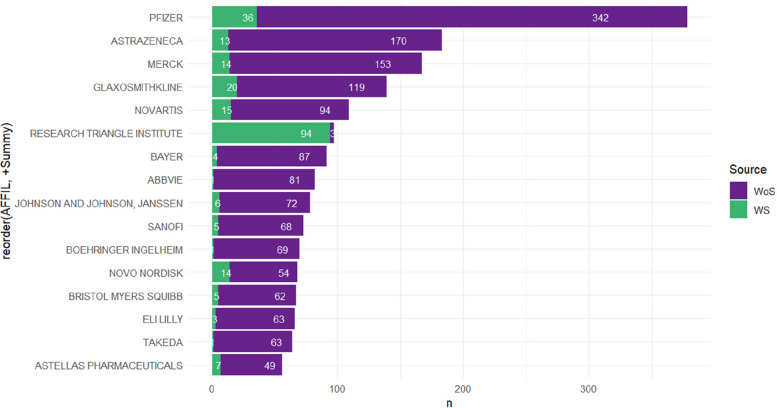
Affiliation and co-affiliations by publication (more than 50 publications)

**Fig. 11:**
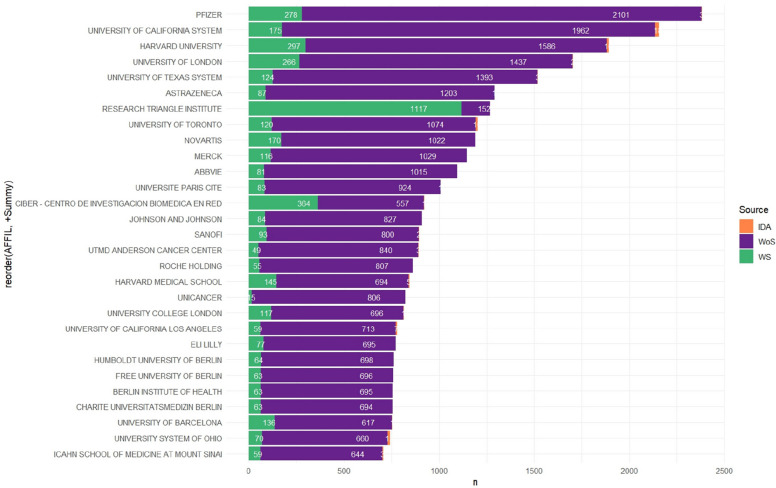
Unique affiliation by publication (more than 700 publications, exclude co-affiliations)

**Fig. 12:**
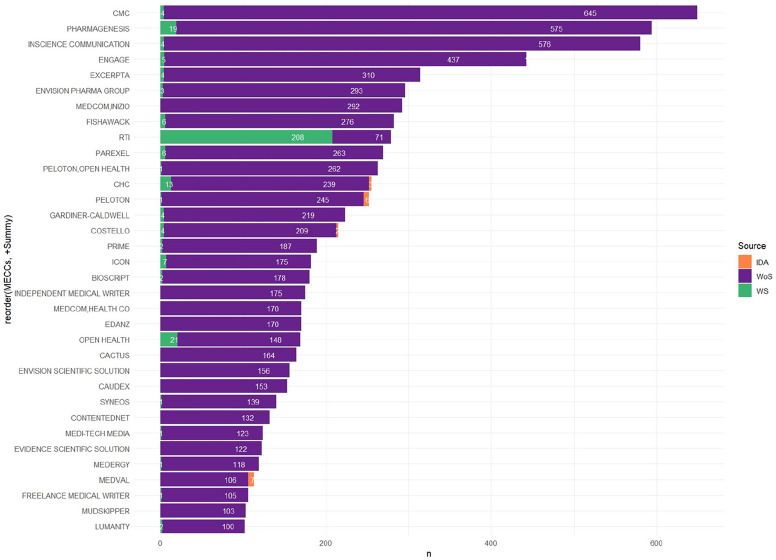
MECC(s) by publication (more than 100 publications)

**Fig. 14:**
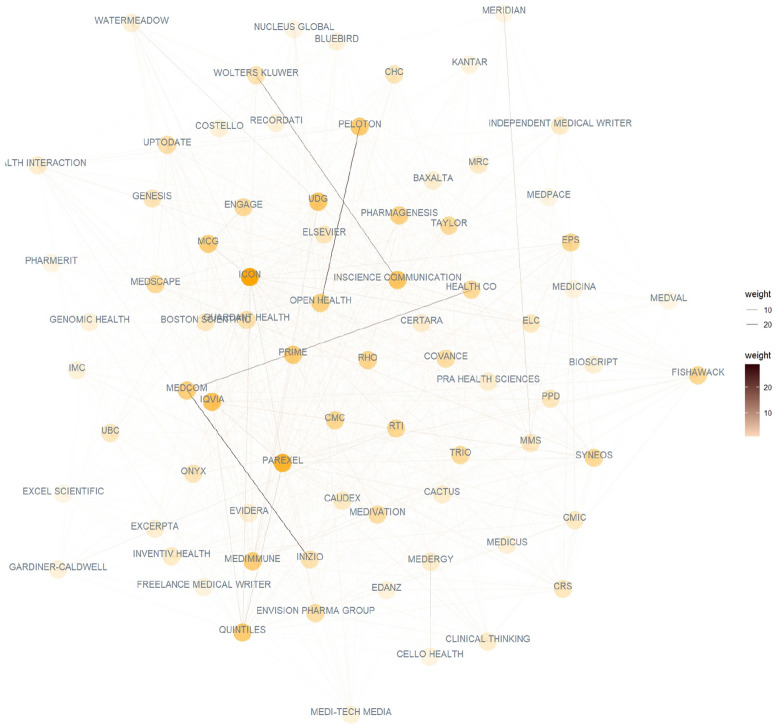
Network of MECCs collaborating on a publication (vertices degree > 25, without duplicates, 76 nodes)

**Fig. 17:**
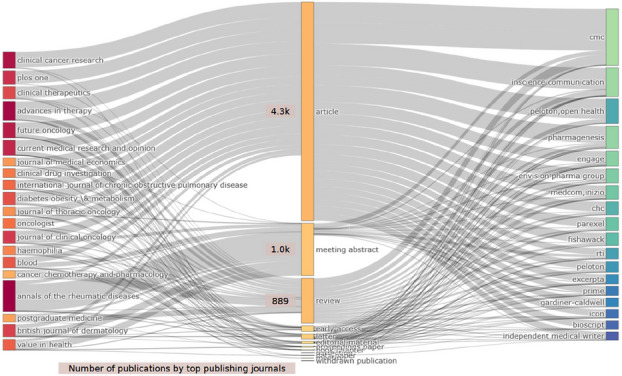
Main types of publication, in relation to the journals that published them and the MECCs acknowledged

## Data Availability

The database is available on Zenodo (https://zenodo.org/records/15537678).

## Appendix 1

### A description of the methods

#### Data collection

This document explains how I built and structured a database of metadata from literature involving medical writing, based on three main sources: Web of Science (WoS), the websites of MECCs, and the Industry Documents Library (IDL).

#### WoS Collection

I used Web of Science to collect metadata about scientific publications that mention medical writing in their acknowledgments. “Technical writ*” or “scientific writ*” also exist but are scarcely used in acknowledgments compared to “medical writ*”, and they can correspond to different services. I thus ran the following search on Web of Science on January 10, 2025:

*"medical writ*" (All Fields) not "medical writ*" (Title) not "medical writ*" (Abstract) not " no medical writ*" (All Fields) not "medical writers were not used" (All Fields) not "WE HAVE NOT USED ANY PROFESSIONAL MEDICAL WRITERS" (All Fields) not "medical writing was not used" (All Fields).*

The query was built based on previous research to find MECCs from the acknowledgments of a similar type of literature. It returned the above list of false positives. While building a reference list of MECCs (Reference List 3, see below), I found additional false positives. I thus excluded items with R that included in their acknowledgments: "DID NOT RECEIVE MEDICAL WRITING" and "NO PROFESSIONAL MEDICAL WRIT".

The number of references collected from WoS is 27 135. The conversion from BibTeX to a dataframe in R and the deletion of duplicate DOIs resulted in a list of 27 100 items. Once I collected the metadata of this literature, I excluded 242 additional false positives by keeping only items including in their acknowledgments “medical writ*”. The total number of items collected from WoS is 26 858.

#### Selecting WoS instead of another platform

WoS was favored over PubMed due to finding a much higher number of publications mentioning medical writing in acknowledgments on WoS.^3^ In addition, PubMed mainly hosts biomedical literature and thus would restrain the possibilities to explore other scientific domains, making use of medical writing.

#### MECC Websites Collection

Some MECCs make their database of articles publicly available (eg, IQVIA). I thus collected the metadata of this literature to supplement the database of commissioned literature collected from WoS. The data collected were limited to the necessary metadata concerning the articles to retrieve DOIs. I used data scraping tools when the number of documents was higher than a few hundred documents, which would be too long to retrieve manually. I used two R libraries to collect these data:

- Rvest 1.0.4 relies on HTML or CSS tags of a website. The main inconvenience is that the code has to be adapted to each website because HTML and CSS codes vary from website to website.
- Rselenium 1.7.9 was helpful when a website loads JavaScript, which Rvest cannot bypass.

**Table 2:** List of references and DOIs found on MECCs websites

DATA source	#References found	#DOIs found in the references	#DOIs indexed in WoS
*Total*	*14,455*	*5493*	*4567*
**RTIHS**	8612	3268	2709
**Open health**	678	623	557
**IQVIA**	3599	571	519
**COS**	719	481	361
**Cellworks**	156	139	90
**GKM**	149	92	80
**Kstrategic**	48	48	47
**RBCCons**	116	70	47
**KJT**	57	53	48
**Alcedis**	43	38	32
**MC Analytics**	38	36	17
**Costello**	180	17	17
**Boston**	20	20	16
**Gouya Insights**	19	17	15
**Csur**	21	20	12

The conversion to a dataframe resulted in a database of 4 555 items.

*Data source:* The source of the data is the websites of the companies listed in this column.

Reference found: I went through the websites to collect the references listed there.

*DOIs found in the references:* To find the DOIs of the documents cited, I first searched if the DOIs were explicitly mentioned in the list of documents collected from the websites of the companies listed in the table above. Concretely, I applied the following regular expression to each reference I had collected about a publication: "10.\d{4,9}/[-._;()/:a-z0-9A-Z] + " (in RStudio), which was found to match 97% of DOIs.^4^ However, it left dots at the end of the DOIs, which I then cleared with an additional regular expression.

*DOIs indexed in WoS:* Once I had a list of DOIs, I searched for them in WoS and collected the BibTeX files of those DOIs (on January 10, 2025). IQVIA suffered the largest loss. It is partly due to the type of literature shared, which included a large number of posters, for example.

#### Limitations

Semi-automatic methods of data collection, especially for RTI and IQVIA, were designed to extract DOIs, in case the website did not provide the DOI of an article or an abstract, it was automatically excluded from the dataset. Another limitation concerns Web of Science: If the DOI of a MECC publication was not found in WoS, then the publication was not included in the dataset. Another limitation concerns the IQVIA and COS databases, which only made articles published until 2023 public (only one article published in 2024 was available).

I also excluded content such as blog posts or opinion pieces found on the Costello Medical websites, and literature about scientific communications or medical writing (eg, Trilogy). Finally, I excluded the literature when only a small subset was available, such as a selection of the most recent publications of a MECC (eg, Generativity Health Outcomes Research).

#### Industry Documents Library Collection

I collected publications from publication trackers of several companies found on the Industry Documents Library (IDL) from October 04 to November 25, 2024. To collect these documents, I first had to find publication trackers: I searched for “publication*”, selected “spreadsheet” and hid possible duplicates (10 April 2024). It resulted in 4323 results. After scanning 1000 results, they appeared to be irrelevant. I then searched for “manuscript*” track*, selected spreadsheet and hid possible duplicates (10 April 2024), which displayed 300 results, and contained relevant results. I selected the five most recent publication trackers among the ones I found. Here is the list:

**Table 3:** List of references and DOIs found in the Industry Documents Library

Source of the document	References found in the IDL	DOIs found in the references	DOIs indexed in WoS
*TOTAL*	***726***	***298***	***229***
McKinsey (Purdue)	51	44	37
TEVA	125	34	15
JUUL	24	6	5
Mallinckrodt	388	185	146
Philip Morris	138	29	26

The list of DOIs was retrieved manually. The publication trackers often contain titles, authors’ names, publication status and publication dates (if published). I searched for this information in Google, Crossref and PubMed to find the corresponding DOIs. If the DOI was not found (either it did not exist or it was impossible to find), I coded it as “not found”. Using the DOIs of this literature, I collected its metadata from WoS on January 13, 2025.

#### Limitations

I found errors in the publication trackers. In addition, the references listed in those documents sometimes have a work-in-progress status, and the title had changed by the time it was published. Because abstracts often are followed up with the publication of the corresponding article, I sometimes found the article and the abstract, but I only included the article, not both.

I only collected the literature from the publication trackers, even though some articles or drafts can be found in the IDL. The publication tracker from McKinsey was the least clear in terms of involvement of Purdue or McKinsey in the production of the literature listed in their publication tracker. Because it was a publication tracker, I still included this literature in the dataset (37 publications).

#### Data cleaning and harmonization

Once I had collected the metadata of the DOIs I had found from the MECCs websites, WoS, and the IDL, I cleaned and harmonized it (see Appendix 2).

#### From BibTeX to Bibliometrix

I collected the BibTeX files of the DOIs of the publications listed above from Web of Science. I then transformed these BibTeX files into a dataframe (RStudio) with the function convert2df() from the library Bibliometrix 4.3.2. This function led to data loss, and *NAs* were found in the column of the DOIs (from the WoS collection). Another sporadic issue when producing the dataframe with convert2df() consisted of errors in data distribution, and some were wrongly categorized. Once in Bibliometrix, I removed all duplicate DOIs from each dataset with the function distinct() from the library dplyr 1.1.2.

In the final dataset, the WS collection includes 4 555 DOIs; the WoS collection includes 26 858 DOIs; and the IDL collection includes 229 DOIs. The final database includes 31 642 items, and 29 911 unique items. Overlaps exist among these databases. I could identify these overlaps by searching similar DOIs in those databases. However, some DOIs in the WoS are missing, which is a limitation to this method.

I then harmonized several categories of data, which I needed for the analysis: publishers, funders, affiliations, and MECCs (see Appendix 2). Publishers could be manually harmonized due to a rather clean list, in contrast with the rest of the data, for which I had to develop reference lists in addition to harmonizing those lists. These processes are described below.

## Results

### List of funders: Reference List 1

#### Cleaning

In the final list of the data collected from WoS, funders needed to be harmonized: Sometimes, they appeared as stand-alone, and sometimes, with the affiliations of the authors, which often included universities, networks, collaborations, consortia or programmes, which I removed. A company could also be cited together with its geographical location (eg, Novartis, India). In this case, I removed the location to keep only the company name. The redundancy of funders and alternative ways of writing the name of a company was partly cleaned manually. I kept the names as short as possible to cover as many mentions as possible. When the names triggered false positives, I removed those triggers (eg, the city names related to “Shire”).

#### Reference List

I used str_extract_all() (from the package stringr 1.5.1) with reference list 1 to extract the matching patterns in the column of funders in the database.

## Results

### List of affiliations: Reference List 2

#### Cleaning

Affiliations are better harmonized than funders in the WoS metadata, and I manually cleaned clear redundancy and errors. I did not harmonize different references to a university, whether they refer to the university in general, to the department belonging to this university, or to the geographic affiliation of that university.

Companies were treated differently, because alternative ways of writing follow different patterns (except sometimes geographically). For example, I deleted the types of companies (AB, inc., etc.). Names also sometimes change according to the department of the companies. However, I cannot treat departments of other organizations (like companies or NGOs) like departments of universities, which are bound to freedom of research, while companies have global strategies and implement them at local levels. I thus left “DALIAN MEDICAL UNIVERSITY” and “DALIAN UNIVERSITY,” for example. I also left hospitals when founded by the Red Cross, for example, but harmonized departments of the Red Cross as “RED CROSS”. Similarly, Samsung was distinguished from Samsung Medical Center because it is a hospital. Additional noise was also deleted (eg, “ABB” or “RHO”).

#### Reference List

I used the same method as for the “funders” reference list to harmonize affiliations. Once I had a cleaned list of affiliations, I used str_extract_all() (from the package stringr 1.5.1) with reference list 2 to extract the matching patterns in the affiliations column in the database.

## Results

### List of MECCs: Reference List 3

#### Step 1

I built a list of MECCs based on previous research, which gathered a list of MECCs found in the acknowledgments of commissioned literature indexed in Web of Science, on LinkedIn and through web searches. I manually added the MECCs that appeared in the IDL, acknowledgments collected from the WoS and MECCs websites if they were missing from the acknowledgments (eg, CSUR).

#### Step 2

Once imported in R, I could visualize and then harmonize the list and merge MECCs together, when they were the same. This list of MECCs was used as a reference list to automatically search in acknowledgments if a MECC was mentioned. To do so, I used str_extract_all() (from the package stringr 1.5.1) to extract the matching patterns in the acknowledgments column in the database and in the list of MECCs. I excluded pharmaceutical companies from the list of MECCs because funders are often cited in the acknowledgments of articles, and it would introduce a significant bias. I also removed universities, plurals, and duplicates.

### Refining the reference lists

#### Step 3

After making a first list and running str_extract_all() to apply the reference lists on the lists of MECCs and funders, I extracted 4 698 items where MECC and funders were missing, even though “medical writ” was mentioned in the acknowledgments. I then went through these items manually to retrieve missing funders and MECCs and spot potential errors to improve the corresponding reference lists.

Finally, I also identified additional false positives (see the section about data collection from WoS) and added a grepl filter (R library dplyr 1.1.4) to exclude them from the final dataset.

#### Step 4

I applied the final reference list on the database. The visualization flagged additional false positives, which concerned companies included in the reference lists (eg, CORE). The names were verified by searching for a specific word in the data and reviewing what word corresponded to the search. For example, a funder is called “AVID” and returned 33 results. After identifying the sentences where it appeared (in the acknowledgments), 22 results referred in fact to “DAVID” and 3 to “AVIDITY”. The word “AVID” could thus be removed from the reference list of funders to avoid false positives, even though the number of hits was too small to significantly affect the final results. I also verified if names were not geographical names. One exception was the company “Galapagos,” which often appears in the dataset, but it clearly refers to the company and not to the islands.

Note that searching by MECCs in the “Acknowledgments” yields more results (868 of the 4784 publications coming from the IDL and the MECCs websites) than searching with queries related to medical writing (252 publications of the 4784 publications). In other words, the MECC reference list provides better results.

## Results

**MECCs and Funders**

The range of this dataset is 226 (227–1). This figure shows most MECCs appeared only once in the acknowledgments of a publication funded by a particular funder.

The range of this dataset is 334 (335–1). This figure shows most funders only funded once a publication acknowledging one particular MECC.

In summary, Figure 15 and Figure 16 show that a small number of funders funded a small number of MECCs through publications.

### Publications

**Table 4:** Types of publication by number of publication

Types of publications	n
ARTICLE	22 512
REVIEW	4681
MEETING ABSTRACT	1211
LETTER	510
ARTICLE; EARLY ACCESS	419
EDITORIAL MATERIAL	298
ARTICLE; PROCEEDINGS PAPER	107
REVIEW; EARLY ACCESS	64
CORRECTION	28
ARTICLE; BOOK CHAPTER	22
LETTER; EARLY ACCESS	13
REVIEW; BOOK CHAPTER	9
ARTICLE; DATA PAPER	7
EDITORIAL MATERIAL; EARLY ACCESS	7
MEETING	5
NOTE	4
PROCEEDINGS PAPER	4
ARTICLE; RETRACTED PUBLICATION	3
BOOK	2
NEWS ITEM	2
ARTICLE; PUBLICATION WITH EXPRESSION OF CONCERN	1
ARTICLE; WITHDRAWN PUBLICATION	1
BOOK REVIEW	1

Biomedical research often starts with the presentation of findings at a conference, and thus, with a meeting abstract. It is thus expected that a fair amount of abstracts (if they receive a DOI) exist in this database.

Figure 17 shows that meeting abstracts are greatly represented in the publications in the *Annals of the Rheumatic Diseases,* which is also the journal that is a top publisher of the literature in the database.

Except for this journal, there seems to be a fair distribution of the type of publication by MECCs and journals.

### Journals

Visualizing publications by journals offers insights on the topics of interest of the MECCs and more generally on the funders, and if some journals are regularly targeted.

**Publishers**

Figure 19 shows how many publications were published by publishers, which are categorized by ownership (eg, all companies belonging to Elsevier are signaled with a black dot), and divided by source of data collection. Considering publications by different company names or by parent company changes the order of the publications’ share. However, it is clear that top publishers of this literature are Wiley, Elsevier, Springer, and Taylor and Francis.

#### Limitations

One of the challenges to harmonize the funder, affiliation and MECC lists is due to the frequent rebranding and merging of these companies. It is impossible to track all companies and their subsidiaries in the world, especially when they were re-branded, sold or bought.

The reference list method with str_extract_all() has limitations. Funders could not be included when their names were written incorrectly. Also, names can be written either in native language or translated in English (eg, French National Research Agency (ANR) can also be written “Agence nationale de la recherche”). These reference lists do not include translations. Sometimes acronyms and full names are written together, sometimes not. Thus, the reference list includes the full name and the acronym, which I could then merge in R with gsub() (see Appendix 2).

This method failed to identify when pharmaceutical companies use in-house medical writing. Sometimes, a mention refers to it though (eg, “Amrit, the ASIA PACIFIC MEDICAL WRITING TEAM of SANOFI-AVENTIS” was mentioned in one article).

Selecting MECCs through acknowledgment relies on the self-reporting of funding, acknowledgments and affiliations, whose information can be erroneous or omitted. Also, a few acknowledgments were found in the list of funders (the problem is either located in the BibTeX dataset downloaded from WoS or during the conversion of the BibTeX to a dataframe by Bibliometrix).

#### Analytical tools

Threefieldsplot (from Bibliometrix) has its own way of harmonizing data, and while I used the categories of data cleaned with dictionaries for MECCs and funders, the data cleaning process of this function seems to perform differently than the method with dictionaries for the affiliation. I used the original category for affiliations when using this function. Also, because results do not include comparison among sources, I applied this tool on the database without duplicate DOIs.

## Appendix 2

### Harmonization lists

*MECCs*

# Harmonize Name companies.

dfdf$MECCs <—gsub('2THENTH','2 THE NTH',df$MECCs).

df$MECCs <—gsub('4CLINICS','4 CLINICS',df$MECCs).

df$MECCs <—gsub('ALPHASCIENTIA','ALPHA SCIENTIA',df$MECCs).

df$MECCs <—gsub('ALPHAR-MAXIM','ALPHARMAXIM',df$MECCs).

df$MECCs <—gsub('APOTHE COM','APOTHECOM',df$MECCs).

df$MECCs <—gsub('BIO CON-NECTIONS','BIO CONNECTION',df$MECCs).

df$MECCs <—gsub('BRIEFINGSTUDIO','BRIEFING STUDIO',df$MECCs).

df$MECCs <—gsub('CE-MEDICAL','CE MEDICAL WRITING',df$MECCs).

df$MECCs <—gsub('CIRCLESCIENCE','CIRCLE SCIENCE',df$MECCs).

df$MECCs <—gsub('CLINICALTHINKING','CLINICAL THINKING',df$MECCs).

df$MECCs <—gsub('COMPLETE HEALTHVIZION|COMPLETE HEALTH-VIZION|COMPLETE HEALTH VIZION','COMPLETE HEALTH VIZION',df$MECCs).

df$MECCs <—gsub('COMPLETE HEALTHCARE','CHC',df$MECCs).

df$MECCs <—gsub('CONTENT\'ED NET|CONTENT ED NET','CONTENTEDNET',df$MECCs).

df$MECCs <—gsub('DP-MEDSYSTEMS','DPMEDSYSTEMS',df$MECCs).

df$MECCs <—gsub('ECC-ONCOLOGY','ECCONCOLOGY',df$MECCs).

df$MECCs <—gsub('EDIT INC','EDIT, INC',df$MECCs).

df$MECCs <—gsub('ELM-COM','ELMCOM',df$MECCs).

df$MECCs <—gsub('ETHIS INC','ETHIS, INC',df$MECCs).

df$MECCs <—gsub('FIRE KITE','FIREKITE',df$MECCs).

df$MECCs <—gsub('H + O COMMUNICATION','H + O COMMUNICATION',df$MECCs).

df$MECCs <—gsub('HEALTH INTER- ACTIONS','HEALTH INTERACTION',df$MECCs).

df$MECCs <—gsub('IN VENTIV MEDICAL|IN-VENTIV HEALTH','INVENTIV HEALTH',df$MECCs).

df$MECCs <—gsub('IN SCIENCE COMMUNICATION','INSCIENCE COMMUNICATION',df$MECCs).

df$MECCs <—gsub('INTER-COMM','INTERCOMM',df$MECCs).

df$MECCs <—gsub('JK MEDICAL COMMUNICATION|JK ASSOCIATES','JK ASSOCIATES, FISHAWACK',df$MECCs).

df$MECCs <—gsub('KNOWLEDGEPOINT360','KNOWLEDGEPOINT 360',df$MECCs).

df$MECCs <—gsub('LOCK-WOOD','LOCKWOOD GROUP',df$MECCs).

df$MECCs <—gsub('MA.CRO','MA-CRO LIFESCIENCE',df$MECCs).

df$MECCs <—gsub('MEDITECHMEDIA|MEDITECH MEDIA|MEDI TECH MEDIA','MEDI-TECH MEDIA',df$MECCs).

df$MECCs <—gsub('NUVO-GROUP','NUVOGROUP',df$MECCs).

df$MECCs <—gsub('PRA INTERNATIONAL','PRA HEALTH SCIENCES',df$MECCs).

df$MECCs <—gsub('PRECISIONSCIENTIA|PRECISION HEALTH ECONOMICS|PRECISION SCIENTIA|PRECISION VALUE AND HEALTH','PRECISION MEDICINE GROUP',df$MECCs).

df$MECCs <—gsub('RTI H|RTI INTERNATIONAL|RTI-HEALTH|RTI-HS|RTIHS|RESEARCH TRIANGLE','RESEARCH TRIANGLE INSTITUTE',df$MECCs).

df$MECCs <—gsub('SUCCINCT CHOICE MEDICAL COMMUNICATION|SUCCINCT HEALTHCARE','SUCCINCT MEDICAL COMMUNICATIONS',df$MECCs).

df$MECCs <—gsub('TEAM9 SCIENCE|TEAM9SCIENCE','TEAM 9 SCIENCE',df$MECCs).

df$MECCs <—gsub('OPEN VIE','OPEN HEALTH',df$MECCs).

df$MECCs <—gsub('OPEN HEALTH,OPEN HEALTH|OPEN HEALTH,OPEN HEALTH,OPEN HEALTH','OPEN HEALTH',df$MECCs).

df$MECCs <—gsub('OPEN HEALTH,OPEN HEALTH','OPEN HEALTH',df$MECCs).

df$MECCs <—gsub('CMC, MCCANN|CMC, IPG|COMPLETE MEDICAL','CMC',df$MECCs).

df$MECCs <—gsub('INSCIENCE COMMUNICATION,SPRINGER','INSCIENCE COMMUNICATION',df$MECCs).

df$MECCs <—gsub('SPRINGER','INSCIENCE COMMUNICATION',df$MECCs).

df$MECCs <—gsub('INSCIENCE COMMUNICATION, INSCIENCE COMMUNICATION','INSCIENCE COMMUNICATION',df$MECCs).

df$MECCs <—gsub('OXFORD PHARMAGENESIS','PHARMAGENESIS',df$MECCs).

df$MECCs <—gsub('UNITED BIOSOURCE CORPORATION','UBC',df$MECCs).

df$MECCs <—trimws(df$MECCs).

df$MECCs <—gsub('RESEARCH TRIANGLE INSTITUTE','RTI',df$MECCs).

df$MECCs <—gsub("RTI,RTI", "RTI", df$MECCs).

df$MECCs <—gsub("CIRI", "", df$MECCs).

df$MECCs <—gsub("COD", "", df$MECCs).

df$MECCs <—gsub("BIOSCIENCE", "", df$MECCs).

*Funders*

# # # Harmonize name Funders.

df$FUND <—gsub('ALK ABELLO','ALK-ABELLO',df$FUND).

df$FUND <—gsub('ALLENAND HANBURY\'S','ALLEN AND HANBURY\'S',df$FUND).

df$FUND <—gsub('ARJO HUNTLEIGH','ARJO INC',df$FUND).

df$FUND <—gsub('ASTRA ZENECA','ASTRAZENECA',df$FUND).

df$FUND <—gsub('ASTRA-ZENECA','ASTRAZENECA',df$FUND).

df$FUND <—gsub('BERLIN CHEMIE','BERLIN-CHEMIE',df$FUND).

df$FUND <—gsub('BMS','BRISTOL MYERS SQUIBB',df$FUND).

df$FUND <—gsub('SQUIBB','BRISTOL MYERS SQUIBB',df$FUND).

df$FUND <—gsub('BRISTOL MYERS BRISTOL MYERS SQUIBB','BRISTOL MYERS SQUIBB',df$FUND).

df$FUND <—gsub('CANADIAN INSTITUTE FOR HEALTH','CANADIAN INSTITUTE OF HEALTH',df$FUND).

df$FUND <—gsub('CANTON GOVERNMENT','CANTON\'S GOVERNMENT',df$FUND).

df$FUND <—gsub('CDC','CENTERS FOR DISEASE CONTROL',df$FUND).

df$FUND <—gsub('CSD MR','CSD MEDICAL RESEARCH',df$FUND).

df$FUND <—gsub('DICKINSON','BD CANADA',df$FUND).

df$FUND <—gsub('BECTON','BD CANADA',df$FUND).

df$FUND <—gsub('DOHME|MSD','MERCK',df$FUND).

df$FUND <—gsub('EU COMMISSION','EUROPEAN COMMISSION',df$FUND).

df$FUND <—gsub('FOOD AND DRUG ADMINISTRATION','FDA',df$FUND).

df$FUND <—gsub('GSK|SMITH KLINE','GLAXOSMITHKLINE',df$FUND).

df$FUND <—gsub('HEXAL','HEXAL, SANDOZ',df$FUND).

df$FUND <—gsub('DEPARTMENT OF HEALTH AND HUMAN SERVICES','HHS',df$FUND).

df$FUND <—gsub('INNOVATIVE MEDICINE INITIATIVE','INNOVATIVE MEDICINES INITIATIVE',df$FUND).

df$FUND <—gsub('ISCIII','CARLOS III',df$FUND).

df$FUND <—gsub('JANSEN','JANSSEN',df$FUND).

df$FUND <—gsub('JUVENILE DIABETES RESEARCH FOUNDATION','JDRF',df$FUND).

df$FUND <—gsub('KITE','GILEAD',df$FUND).

df$FUND <—gsub('LEO LABORATORIES','LEO PHARM',df$FUND).

df$FUND <—gsub('MARIE CURIE','MARIE SKLODOWSKA-CURIE',df$FUND).

df$FUND <—gsub('MILLENIUM','MILLENNIUM',df$FUND).

df$FUND <—gsub('NETHERLANDS ORGANISATION FOR HEALTH RESEARCH|ZONMW','NETHERLANDS ORGANIZATION FOR HEALTH RESEARCH',df$FUND).

df$FUND <—gsub('NATIONAL INSTITUTE OF ALLERGY','NIAID',df$FUND).

df$FUND <—gsub('NATIONAL INSTITUTE OF ARTHRITIS','NIAMS',df$FUND).

df$FUND <—gsub('NATIONAL INSTITUTE OF CHILD HEALTH','NICHD',df$FUND).

df$FUND <—gsub('NATIONAL INSTITUTE ON DRUG ABUSE','NIDA',df$FUND).

df$FUND <—gsub('NATIONAL INSTITUTE OF DENTAL AND CRANIOFACIAL RESEARCH','NIDCR',df$FUND).

df$FUND <—gsub('NATIONAL INSTITUTE OF DIABETES','NIDDK',df$FUND).

df$FUND <—gsub('NATIONAL INSTITUTE OF DISABILITY','NIDILRR',df$FUND).

df$FUND <—gsub('NATIONAL INSTITUTE ON DISABILITY','NIDRR',df$FUND).

df$FUND <—gsub('NATIONAL INSTITUTE OF ENVIRONMENTAL HEALTH SCIENCES','NIEHS',df$FUND).

df$FUND <—gsub('NATIONAL INSTITUTE OF FOOD AND AGRICULTURE','NIFA',df$FUND).

df$FUND <—gsub('NATIONAL INSTITUTE OF GENERAL MEDICAL SCIENCES','NIGMS',df$FUND).

df$FUND <—gsub('NATIONAL INSTITUTE FOR HEALTH RESEARCH','NIHR',df$FUND).

df$FUND <—gsub('NATIONAL INSTITUTE OF HEALTH|NIH \(|NIH;NIH)|NATIONAL INSTITUTES HEALTH|NIH ','NATIONAL INSTITUTES OF HEALTH',df$FUND).

df$FUND <—gsub('NATIONAL INSTITUTE OF MENTAL HEALTH','NIMH',df$FUND).

df$FUND <—gsub('NATIONAL INSTITUTE OF NEUROLOGICAL DISORDERS','NINDS',df$FUND).

df$FUND <—gsub('NATURAL SCIENCE FOUNDATION','NSF',df$FUND).

df$FUND <—gsub('PROCTOR GAMBLE','PROCTER',df$FUND).

df$FUND <—gsub('POLICY RESEARCH PROGRAMME','PRP',df$FUND).

df$FUND <—gsub('SEAGEN','SEAGEN, PFIZER',df$FUND).

df$FUND <—gsub('SHIRE DEVELOPMENT|SHIRE PHARMACEUTICAL|SHIRE INTERNATIONAL|SHIRE HUMAN GENETIC THERAPIES|SHIRE GROUP|SHIRE LLC|SHIRE (A TAKEDA COMPANY)|SHIRE HGT|SHIRE, ZUG','SHIRE, A TAKEDA COMPANY',df$FUND).

df$FUND <—gsub('SALIX','SALIX, VALEANT',df$FUND).

df$FUND <—gsub('SWISS NATIONAL SCIENCE FOUNDATION|SNSF','SNF',df$FUND).

df$FUND <—gsub('SWEDISH ORPHAN BIOVITRUM','SOBI',df$FUND).

df$FUND <—gsub('AGENCY FOR HEALTHCARE RESEARCH AND QUALITY','AHRQ',df$FUND).

df$FUND <—gsub('US DEPARTMENT OF AGRICULTURE','USDA',df$FUND).

df$FUND <—gsub('LABORATORIES VICHY|VICHY LAB','LABORATOIRES VICHY',df$FUND).

df$FUND <—gsub('MERCK,MERCK|MERCK,KENILWORTH','MERCK',df$FUND).

df$FUND <—gsub('GLAXOSMITHKLINE,GLAXOSMITHKLINE','GLAXOSMITHKLINE',df$FUND).

df$FUND <—gsub('GENZYME','SANOFI',df$FUND).

df$FUND <—gsub('SANOFI,SANOFI','SANOFI',df$FUND).

df$FUND <—gsub('SANOFI,REGENERON,SANOFI','SANOFI,REGENERON',df$FUND).

df$FUND <—gsub('ASTRAZENECA,ASTRAZENECA','ASTRAZENECA',df$FUND).

df$FUND <—gsub("EMC..", "EMC", df$FUND).

df$FUND <—gsub("- BIOSCIENCES", "", df$FUND).

df$FUND <—gsub(". BRAUN", "BRAUN", df$FUND).

*Affiliations*

## Harmonize name affiliations.

df$AFFIL <—gsub('AGENCY FOR SCIENCE TECHNOLOGY AND RESEARCH','A*STAR',df$AFFIL).

df$AFFIL <—gsub('BRISTOL-MYERS SQUIBB','BRISTOL MYERS SQUIBB',df$AFFIL).

df$AFFIL <—gsub('GLAXO SMITHKLINE','GLAXOSMITHKLINE',df$AFFIL).

df$AFFIL <—gsub('ICON GROUP|ICON PLC','ICON',df$AFFIL).

df$AFFIL <—gsub('JOHSON AND JOHNSON','JOHNSON AND JOHNSON',df$AFFIL).

df$AFFIL <—gsub('LILLY USA|LILLY SA|LILLY PHARMACEUTICALS|LILLY DEUTSCHLAND','ELI LILLY',df$AFFIL).

df$AFFIL <—gsub('SCHERING PLOUGH','SCHERING-PLOUGH',df$AFFIL).

*Publishers*

## harmonize name publishers.

df$PU <—gsub('EXCERPTA MEDICA INC|EXCERPTA MEDICA INC-ELSEVIER','EXCERPTA MEDICA, ELSEVIER',df$PU).

df$PU <—gsub('ELSEVIER SCI LTD|ELSEVIER SCIENCE INC','ELSEVIER',df$PU).

df$PU <—gsub('ADIS INT LTD|ADIS INTERNATIONAL LTD','ADIS, SPRINGER',df$PU).

df$PU <—gsub('ACADEMIC PRESS INC ELSEVIER SCIENCE|ACADEMIC PRESS LTD- ELSEVIER SCIENCE LTD','ACADEMIC PRESS, ELSEVIER SCIENCE',df$PU).

df$PU <—gsub('B M J PUBLISHING GROUP|BRITISH MED JOURNAL PUBL GROUP','BMJ PUBLISHING GROUP',df$PU).

df$PU <—gsub('JOHN WILEY AND SONS LTD','WILEY',df$PU).

df$PU <—gsub('NATURE PORTFOLIO','NATURE PORTFOLIO, SPRINGER',df$PU).

df$PU <—gsub('NATURE PUBLISHING GROUP','NATURE PUBLISHING GROUP, SPRINGER',df$PU).

df$PU <—gsub('NATURE RESEARCH','NATURE RESEARCH, SPRINGER',df$PU).

df$PU <—gsub('SAGE PUBLICATIONS INC|SAGE PUBLICATIONS LTD','SAGE',df$PU).

df$PU <—gsub('SPRINGER-VERLAG BERLIN|SPRINGER-VERLAG ITALIA SRL|SPRINGER-VERLAG SINGAPORE PTE LTD','SPRINGER SCIENCE + BUSINESS MEDIA',df$PU).

df$PU <—gsub('SPRINGER VERLAG','SPRINGER SCIENCE + BUSINESS MEDIA',df$PU).

df$PU <—gsub('TAYLOR AND FRANCIS INC|TAYLOR AND FRANCIS LTD','TAYLOR AND FRANCIS',df$PU).

df$PU <—gsub('WILEY-BLACKWELL PUBLISHING, INC','WILEY-BLACKWELL',df$PU).

df$PU <—gsub('WILEY PERIODICALS, INC','WILEY',df$PU).

df$PU <—gsub('OXFORD UNIV PRESS INC','OXFORD UNIV PRESS',df$PU).

df$PU <—gsub('SPRINGER BASEL AG|

SPRINGER FRANCE|SPRINGER HEIDELBERG|SPRINGER INDIA|

SPRINGER INT PUBL AG|SPRINGER INTERNATIONAL PUBLISHING AG|

SPRINGER JAPAN KK|SPRINGER LONDON LTD|SPRINGER TOKYO|SPRINGER WIEN|

SPRINGERNATURE','SPRINGER',df$PU).

## Appendix 3

Lists of Sources.

*The Websites Of MECCs (WS)*

The metadata of the literature found on these websites were collected on January 13, 2025 on WoS and includes 4555 items.

**Table:**

Company	Online source
RTIHS	https://www.rtihs.org/publications
Open health	https://www.openhealthgroup.com/publication-library/
IQVIA	https://www.rwebibliography.com
COS	https://www.clinoutsolutions.com/scientific-contributions
Cellworks	https://cellworks.life/publications?page=0
GKM	https://www.gkm-therapieforschung.de/publikationen/
Kstrategic	https://www.kstrategic.com/
RBCCons	https://www.rbcconsultants.com/publications/
KJT	https://kjtgroup.com/publications-presentations/
Alcedis	https://www.alcedis.de/de/publications
MC Analytics	https://mcanalytics.co.in/publications/
Costello	https://www.costellomedical.com/research/articles-publications/
Boston	https://bostonsp.com/index.php/publications/
Gouya Insights	https://gouya-insights.com/our-work/gouya-insights-publications/
Csur	https://www.csures.com/index.php/publications/

*The Industry Documents Library (IDL)*

The metadata of the literature found in these documents were collected on January 9, 2025 from the WoS and includes 229 items.

**Table:**

Company	Online source
McKinsey (Purdue)	https://www.industrydocuments.ucsf.edu/opioids/docs/#id=mnpj0256
TEVA	https://www.industrydocuments.ucsf.edu/opioids/docs/#id=smkj0326
JUUL	https://www.industrydocuments.ucsf.edu/tobacco/docs/#id=gjgp0326
Mallinckrodt	https://www.industrydocuments.ucsf.edu/opioids/docs/#id=gmcf0247
Philip Morris	https://www.industrydocuments.ucsf.edu/tobacco/docs/#id=mngk0000

## Abbreviations

CRO: Contract research organization
DOI: Digital object identifier
MECC: Medical education and communication company

## Footnotes

1 https://www.industrydocuments.ucsf.edu/

2 https://zenodo.org/records/15537678
